# Grazers extend blue carbon transfer by slowing sinking speeds of kelp detritus

**DOI:** 10.1038/s41598-018-34721-z

**Published:** 2018-11-21

**Authors:** Thomas Wernberg, Karen Filbee-Dexter

**Affiliations:** 1 UWA Oceans Institute & School of Biological Sciences, University of Western Australia, Perth, Australia; 2 Department of Science and Environment, Roskilde University, Roskilde, Denmark; 3 Norwegian Institute for Water Research, Oslo, Norway; 4 Institute of Marine Research, Flødevigen, Norway

## Abstract

Marine plant communities such as kelp forests produce significant amounts of detritus, most of which is exported to areas where it can constitute an important trophic subsidy or potentially be sequestered in marine sediments. Knowing the vertical transport speed of detrital particles is critical to understanding the potential magnitude and spatial extent of these linkages. We measured sinking speeds for *Laminaria hyperborea* detritus ranging from whole plants to small fragments and sea urchin faecal pellets, capturing the entire range of particulate organic matter produced by kelp forests. Under typical current conditions, we determined that this organic material can be transported 10 s of m to 10 s of km. We show how the conversion of kelp fragments to sea urchin faeces, one of the most pervasive processes in kelp forests globally, increases the dispersal potential of detritus by 1 to 2 orders of magnitude. Kelp detritus sinking speeds were also faster than equivalent phytoplankton, highlighting its potential for rapid delivery of carbon to deep areas. Our findings support arguments for a significant contribution from kelp forests to subsidizing deep sea communities and the global carbon sink.

## Introduction

Marine plants are among the most productive primary producers on Earth^1^, forming extensive habitats in the coastal zone^2–4^. The fate of primary production in these coastal habitats has been the focus of ecological studies for decades, and we know a sizeable proportion of their biomass is exported as detritus to adjacent and distant habitats^5–7^. Seminal studies have documented the role of marine plant detritus as a trophic subsidy to ecosystems with low or no primary production e.g.^8–11^. More recently, there has also been a growing interest in the possibility that this material is not consumed but sequestered out of the carbon cycle^12,13^.

Regardless of whether the ultimate fate is consumption or sequestration, important and largely unanswered questions remain concerning the magnitude and transport distances of detritus exported from marine plant communities^13^. These questions are critical because transport distances determine the potential magnitude and spatial extent of trophic subsidy and sequestration. While there have been many observations of deposits of marine plant detritus on the seafloor meters to hundreds of kilometers away from their likely point of origin^14–17^, a comprehensive understanding of detritus dispersal patterns is lacking^13^, at least in part, because transport depends on a complex interplay between waves, currents, topography, and physical characteristics of detrital ‘particles’^18^. With the increasing availability of hydrodynamic particle dispersal (Langrangian) models^19^, e.g.^20^, estimates of possible detritus dispersal pathways are now possible^21^. This enables mapping of carbon transfer pathways, uncovering source-sink dynamics, as well as understanding changes in trophic connectivity or sequestration rates under scenarios of changing ocean currents^19,22,23^ or changing primary production^24–26^. The accuracy of particle dispersal models depends largely on accurate estimates of ocean currents. But, in addition to valid oceanographic parameters, these models require realistic inputs of the speeds at which detrital particles sink (i.e., deposition rates under calm conditions). Accurate estimates of vertical movement are particularly important, because it determines how long particles remain suspended, and therefore how far they can be moved by horizontal currents before they reach the seafloor^27^. Vertical position is also important because of complex spatial and temporal variability in ocean currents. As sinking speeds depend largely on weight, buoyancy, and drag, which is influenced by a range of particle properties including size, shape, and material density e.g.^27^, they are difficult to estimate or calculate for complex structures such as plant detritus. One solution is to determine these empirically.

**Figure 1:**
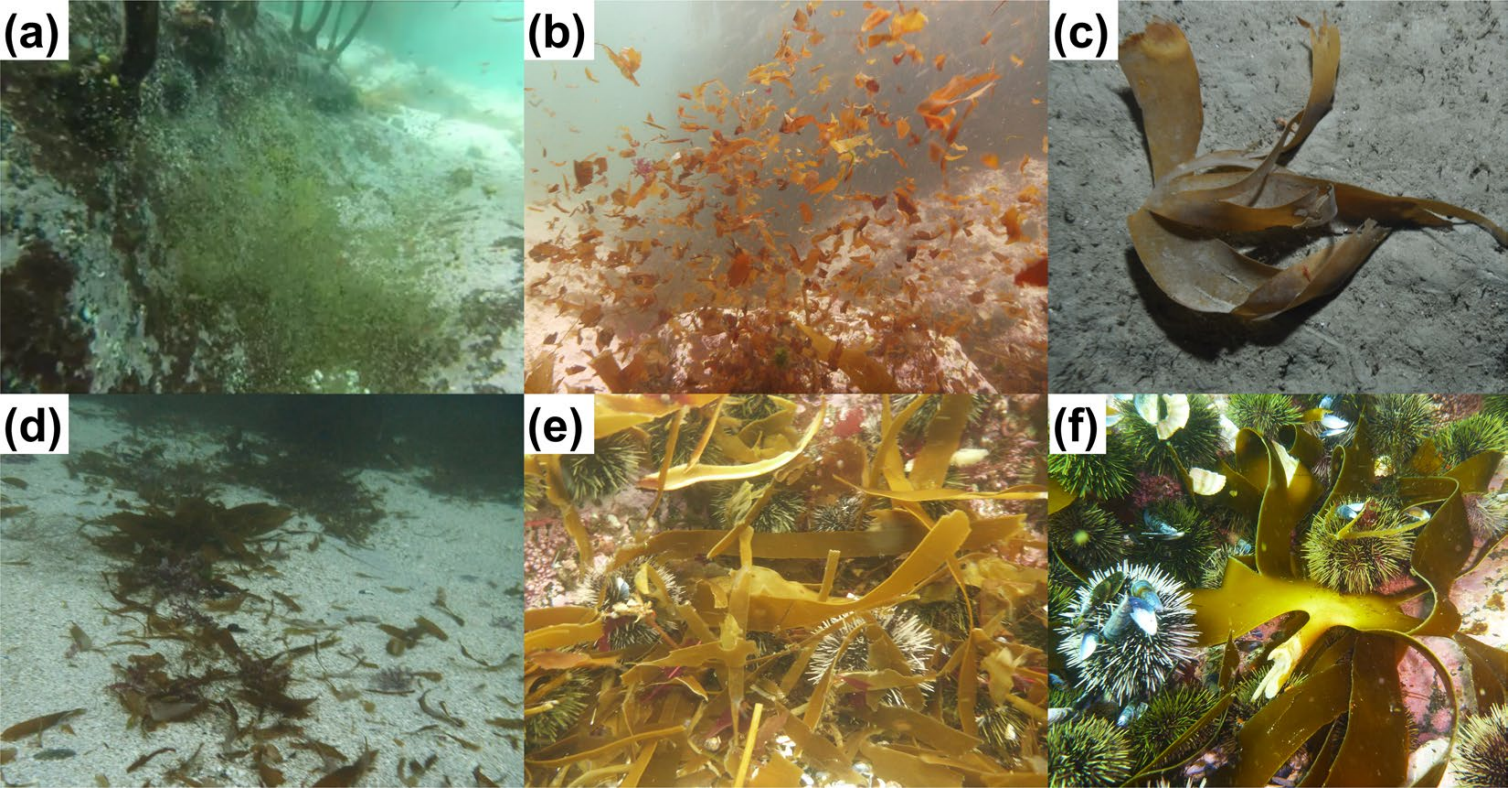
Kelp detrital particles (*Laminaria hyperborea*) from Malangen, northern Norway. (**a**) Accumulation of sea urchin faeces in a small depression, (**b**) small resuspended fragments, (**c**) whole blade at the bottom of the fjord (400 m depth), (**d**) medium sized fragments on a sandy bottom, (**e**) accumulation of fragments attached to, and consumed by, sea urchins (*Strongylocentrotus droebachiensis*), and (**f**) a whole plant being shredded by sea urchins. (Photos: (**c**) K. Filbee-Dexter, all other T. Wernberg).

Kelp forests are highly productive seaweed ecosystems along temperate and Arctic rocky shores^4^. The rates of direct consumption by herbivores are generally low in most kelp forests and on average 80% of the primary production enters the detrital pool^6^, implying a substantial potential for export as a vector of trophic connectivity or sequestration^13^. It has been well documented that a range of processes including dislodgment, tattering and fragmentation by waves and shredding by herbivores generate kelp detrital particles ranging in size from whole plants to small fragments and biogenic pellets (Fig. 1)^17,28–30^. Nevertheless, to our knowledge there have been no published studies of sinking speeds for detritus originating from kelp forests.

*Laminaria hyperborea* is a dominant subtidal kelp throughout the northeast Atlantic that forms extensive forests between 0–25 m depth on rocky coasts from Portugal to northern Norway^31–33^. Approximately 22% of *L. hyperborea* detrital production occurs as distal erosion of small particles and 78% as dislodgment of whole thalli or seasonal loss of old blades (full blades grown over the previous year that are shed during spring)^34^. Large particles can reach deep habitats intact (Fig. 1c) or can be broken down to smaller fragments through abrasion or shredding by herbivores (Fig. 1). *L*. *hyperborea* does not have pneumatocysts or hollow parts so all its detritus sinks. Nevertheless, due to substantial phenological differences in biochemical composition and tissue properties between stipes, new blades, old blades and faeces^35–37^, the sinking speeds likely differ between detritus originating from different thallus origin, size and shape. Here we empirically determine sinking speeds and estimate transport distances for 8 different size and tissue characteristics of *L*. *hyperborea* ranging from sea urchin faeces and fragments to entire blades and plants (Fig. 1).

## Results

Detrital particles measured in this study displayed a broad range of characteristics that will influence their movement in the water column (Table 1). Mean particle area ranged from 0.6 mm^2^ to 0.18 m^2^ and mean biomass ranged from 0.1 mg to 0.65 kg wet weight, capturing the high variability in forms that kelp detritus takes (Fig. 1). Average density of blade material was 1064 ± 96 kg m^−3^ (n = 15) and stipe material was 1288 ± 246 kg m^−3^ (n = 10).

**Table 1:** Characteristics of detrital particles and their sinking speeds measured in this study (mean ± SD [min-max]).

Particle type	Area (mm^2^)	Biomass (g WW)	Sinking speed (m s^−1^)
Small sea urchin faeces (n = 24)	0.6 ± 0.4 [<0.1–1.2]	0.0001 ± 0.00001^#^ [5.0E-6–0.0003]	0.008 ± 0.003 [0.002–0.014]
Medium sea urchin faeces (n = 24)	2.7 ± 1.2 [0.8–5.0]	0.0005 ± 0.0002^#^ [0.0002–0.0007]	0.014 ± 0.004 [0.004–0.020]
Large sea urchin faeces (n = 24)	7.7 ± 2.2 [2.9–11.8]	0.0014 ± 0.0004^#^ [0.0004–0.002]	0.012 ± 0.004 [0.005–0.020]
Small blade fragments (n = 49)	63 ± 42 [15–173]	0.14 ± 0.09^¥^ [0.034–0.387]	0.028 ± 0.005 [0.022–0.041]
Medium blade fragments (n = 44)	2229 ± 929 [625–3624]	4.98 ± 2.08^¥^ [1.40–8.11]	0.041 ± 0.030 [0.008–0.150]
Large blade fragments (n = 44)	12112 ± 9315 [3632–36743]	27.1 ± 20.1^¥^ [8.1–82.2]	0.040 ± 0.025 [0.015–0.100]
Blade new (n = 20)	16059 ± 58214 [12238–285300]	291 ± 106 [152–563]	0.076 ± 0.061 [0.008–0.250]
Blade old (n = 10)	165394 ± 46486 [56498–235136]	413 ± 156 [142–694]	0.073 ± 0.027 [0.049–0.121]
Stipe (n = 20)	40363 ± 51644 [19706–212635]	431 ± 52 [334–526]	0.181 ± 0.048 [0.100–0.333]
Whole thallus (n = 10)	182034 ± 36011 [129785–239187]	645 ± 64 [793–575]	0.165 ± 0.049 [0.067–0.250]

^#^Estimated from total biomass of 48 faecal pellets partitioned according to the relative area of each pellet.

^¥^Estimated from area: weight relationship obtained for a subset of blade fragments.

**Figure 2:**
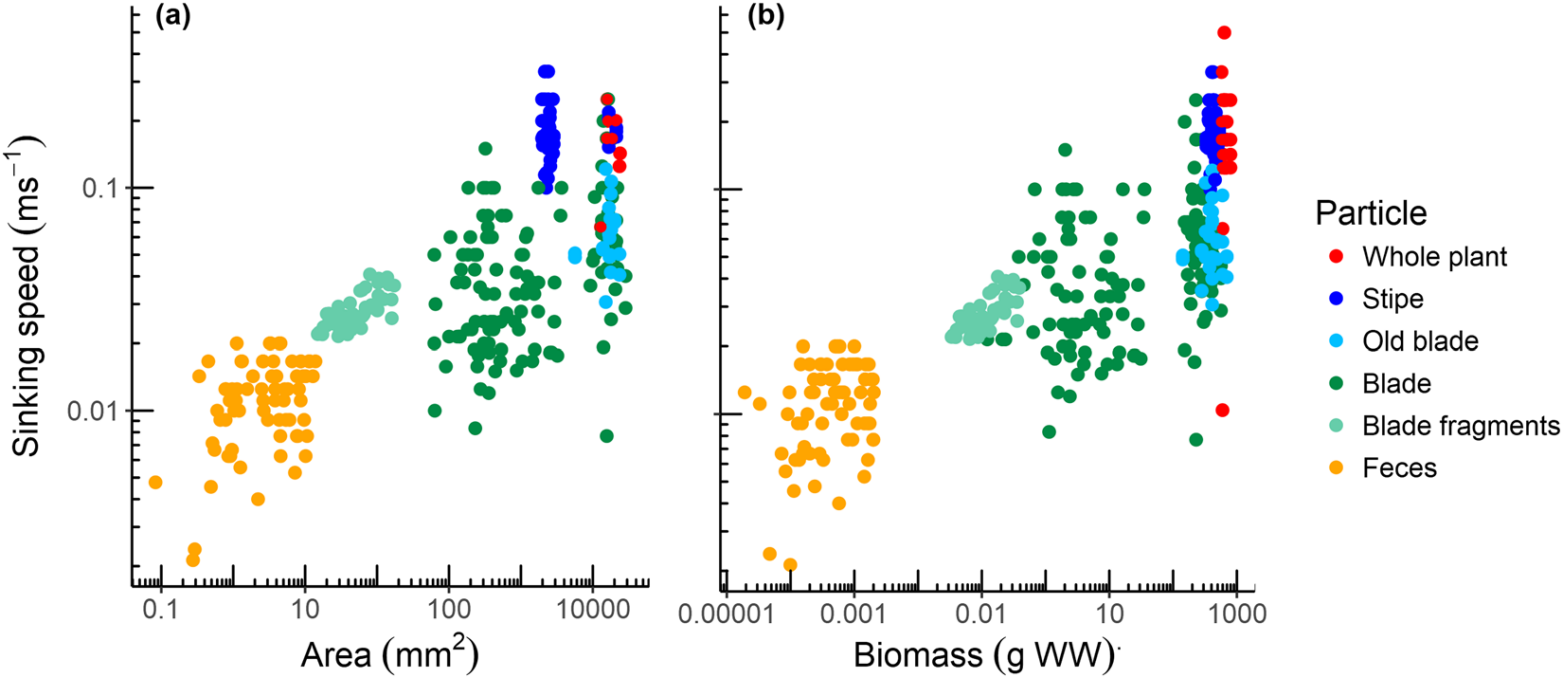
Sinking speeds of different kelp detrital particles against (**a**) area and (**b**) biomass (log scales).

Detrital kelp particles sank at a large range of speeds, from 0.002 m s^−1^ for the smallest particles to 0.5 m s^−1^ for whole plants and stipes (Table 1, Fig. 2). They tended to fall vertically and orient towards maximal downward-facing surface area in the water column (i.e. blades splayed and stipes perpendicular; Supplementary Video), but some particles did move horizontally despite little to no current, the farthest ending up ~20 m away from the drop release position after sinking 4 m depth.

Sinking speed increased with kelp area (ANCOVA, *F*_1,339_ = 125, p < 0.001), but there was high variability with this relationship (Fig. 2), and it depended on the type of kelp material (ANCOVA, *F*_5,339_ = 125, p < 0.001). Stipes and whole plants sank faster compared to other particles (Tukeys posthoc; p < 0.001). There was no strong difference between sinking speeds of old blades, new blades, and blade fragments (Tukeys posthoc; p > 0.05), despite different average biomasses and areas of these particles (Table 1). Faeces sank slower than all other particles (Tukeys posthoc; p < 0.040), with small faeces sinking about half the speed of large and medium sized faeces (Table 1). The highest variabilities in speeds were recorded for whole blades and large and medium blade fragments, and ranged from 0.008 m s^−1^ to 0.25 m s^−1^ (Table 1).

**Figure 3:**
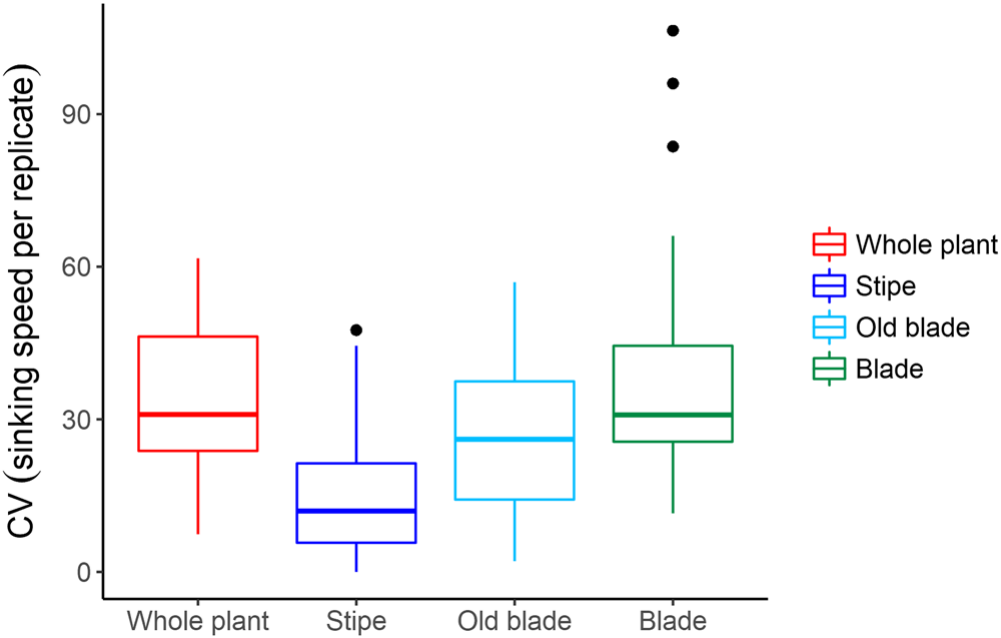
Coefficient of variation (CV) between sinking speeds measured for repeated drops (n = 3) of large particles. Boxes show the lower and upper quartile values and the thick line indicates the median (n = 10 for whole plants and old blades, n = 20 for stipes and blades). The whiskers correspond to 1.5 interquartile range (IQR ~ the 95% confidence interval) and the black dots represent observations outside this range.

Most particles that were measured multiple times did not vary in their sinking speeds among replicate runs (coefficient of variation <40%; Fig. 3). Stipes showed the least variability (apart from one outlier). Blades showed the most variability, with three particles experiencing a large range of sinking rates. This was likely due to their position at release and the extent they compacted, changed shape, and tumbled in the water column (Supplementary Video).

Potential export distances for detrital particles over the range of sinking speeds measured in this study varied from 2 to 940 m if the particle settled at 10 m depth, and from 166 m to 94 km if the particle settled at 1000 m depth (under constant currents, but see^38^). Stipes and whole plants stayed relatively close to their release point, whereas most whole blades and blade fragments settled within 10 s of m to several km from their release point. Sea urchin faeces moved up to a km when settling at 10 m depth and reached 10 s of km when settling at 1000 m depth. Thus export distances of kelp detritus should be greatest in areas with many sea urchins, where currents are strong, or where the sea floor grades quickly to deep depths.

## Discussion

Significant amounts of biogenic carbon are exported from marine plant communities, such as kelp forests, in the form of detritus^6^. How this detritus participates in the global carbon cycle depends on where it ends up (i.e., export distance)^12^. We found a wide range of sinking speeds for kelp particles, showing that the type of particle and the extent of fragmentation or consumption can strongly impact their ultimate fate. Under minimal to moderate current conditions, most blades, stipes, and whole plants will reach the seafloor within a short distance of the kelp forest, whereas small particles and sea urchin faeces can travel substantial distances. This suggests that under these conditions as much as 22%^34^ of kelp-derived carbon could reach the continental shelf and deep sea. These estimates represent lower bounds of maximal potential export distances as they do not account for post-deposition movement or transformation of the detritus (i.e., fragmentation by shredders), nor do they include tidal currents or other locally strong horizontal water movement. In addition, the outlying ‘slow drops’ of blades measured in this study suggest a small portion of large particles has potential to be exported further.

**Figure 4:**
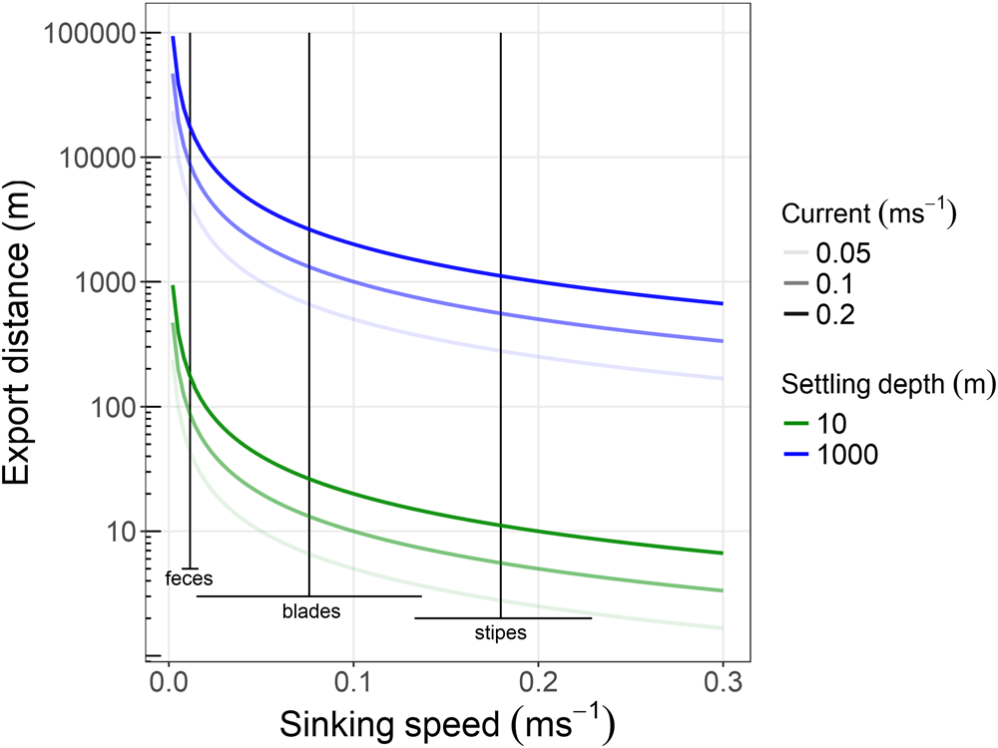
Export distances for detrital particles over the range of sinking speeds measured in this study (slowest sea urchin faeces to fastest stipe), and under different horizontal current speeds. Black lines are average ± SD for faeces, whole blades, and stipes (Table 1). Note: whole thalli have similar sinking speeds as stipes (Table 1).

Sea urchin trapping and grazing of large kelp detritus is a pervasive process in kelp forests globally^25^. A large proportion of the kelp consumed by sea urchins is released as faeces of fresh algal material that has not been digested^35^. Our study demonstrates that sea urchins play a major role in mobilizing kelp detritus by consuming or shredding large particles into small fragments and faeces that sink 20 times slower than whole plants (Table 1). This transformation extends the average detritus dispersal distance 30–50 times (Fig. 4), and so the amount of consumption has implications for the spatial extent of this carbon transfer. Sea urchin grazing intensity on kelp forests has changed dramatically in many regions due to climate change^39–42^. The implied change in consumption rate is likely to have substantially altered the amount of detritus moving through different export pathways, and thus the magnitude and location of detrital deposits.

Detrital kelp particles that are exported large distances can pass into deep depressions, canyons, or cross the continental shelf, eventually reaching the deep sea. In these recipient habitats they may be buried in sediments and contribute to carbon sequestration^12^ or be assimilated by deep benthic communities with little to no *in situ* source of primary production^15,43^. The sinking speeds of small particles in our study of 0.008–0.014 ms^−1^ are 5–10 times higher than the sinking speeds of 0.0013–0.0028 ms^−1^ of similar-sized aggregates of phytoplankton (*Skeletonema costatum* and *Emiliania Huxleyi*, 2.2–4.9 mm^2^)^44^ and were within range of reported sinking speeds of faeces from copepods, macrocrustaceans, tunicates, shrimps, and polychaetes (0.001–0.06 ms^−1^ ^45^). Faeces tend to sink slower and change chemical composition as they degrade and undergo microbial mineralization^45^. The much faster deposition of sea urchin faeces implies less remineralization of the carbon in transit and therefore a greater potential contribution to deep benthic food webs or sequestration relative to phytoplankton derived organic material^13^. Kelps also contain organic molecules that are not easily broken down, which further slows remineralization^46^. Notably, kelp forests often occur in high energy environments^4^ and therefore have a high potential for long distance export that is further extended in areas with many grazers or that grade quickly to deep depths. Our findings are consistent with arguments for a significant contribution from these underwater forests to subsidizing deep sea communities and the global carbon sink.

## Methods

We measured the sinking speeds of 340 kelp (*Laminaria hyperborea*) detrital particles ranging in size from pellets of sea urchin faeces to small blade fragments and whole plants (Table 1). Kelps were collected from Norway (Malangen fjord; 69.63°N 18.01°E) in May 2018 (new blades), cut into pieces matching in size and shape to detritus collected in the field, and photographed and measured for area (Fig. 1) ^17^. To obtain sea urchin faeces, we collected adult green sea urchins (*Strongylocentrotus droebachiensis*) that were directly consuming *L*. *hyperborea*, held them for 24 hours in seawater with unlimited access to *L*. *hyperborea*, and collected fresh faecal pellets with a filter.

Sinking speeds were measured within 24 hours of collection by filming (GoPro Hero3) individual particles of detritus falling through seawater next to a ruler and subsequently calculating sinking distance over video time. Whole plants, blades, stipes and large fragments were measured *in situ* inside a protected marina with no currents by gently releasing them off a jetty next to a 2-m ruler, while small fragments and sea urchin faeces were released through a 20 cm clear pvc pipe (5 cm diameter). Any epiphytes on the kelps were left intact, however, there were no epiphytes on the blades and most stipe epiphytes were almost entirely low-profile encrusting species. Faecal pellets were rinsed in clean seawater to prevent compaction according to methods of Sauchyn and Scheibling^35^. To measure the consistency of sinking rates of large pieces (blades, stipes and whole thalli), we tagged these detrital particles with unique numbers and dropped them off the jetty 3 times (4 times for whole thalli). We dropped a total of 10 whole plants, 20 stipes, 20 new blades and 10 old blades.

To measure particle area and weight, all detrital particles were laid out and photographed against a ruler on a light background and their area (A) measured in Image-J 1.52a (https://imagej.nih.gov/). Whole thalli, stipes and blades were all weighed individually to 0.1 g wet weight (WW). For blade fragments, a subset (n = 15) were weighed. There was a strong linear relationship between blade fragment area and biomass (WW(g) = 0.001 × A(mm^2^) – 1.1814; Pearson’s r^2^ = 0.924, n = 15), so we applied a linear regression to all remaining fragments to determine their biomass.

Material density was determined for a subset of stipes (10) and new blades (15). Stipe density was calculated using wet weight × volume^−1^, using displacement volume measured in water. Average stipe volumes were 0.42 ± 0.08 L. Blade density was determined using wet weight × (area × average thickness)^−1^, using thickness measured at the base (1.29 ± 0.74 mm) and distal ends (0.56 ± 0.13 mm) with venier calipers (mean ± SD).

Sea urchin faeces were classified as small, medium or large size fractions, based on video observations. Faeces that were just large enough to be resolved in the video were classified into the smallest size fraction, faeces with diameters approximately an order of magnitude larger than most were grouped into the largest size fraction, and all others were classified as medium sized. A subset (n = 48) of faeces from across these 3 size categories were photographed on white paper to determine area. To obtain a coarse estimate of biomass (which was challenging with a 0.001 g scale), we weighed all 48 faeces at once, and portioned out the total weight using the relative areas of each fragment. Faeces used to measure sinking speeds were assigned a particle area by randomly selecting an area from the distribution of measures for each size class (assuming a normal distribution). This enabled us to visualize the variability in biomass and area for this size category of detritus, and corresponded well with the size classes of *S. droebachiensis* faeces reported by Sauchyn and Scheibling^47^.

We used an ANCOVA to test the effect of different particle types on sinking speed, using particle area as a covariate. Data met assumptions of normality and heteroscedasticity. Posthoc comparisons were performed with Tukeys HSD test. Analyses were performed in R version 3.5.0.

To investigate the implications of the observed sinking speeds on the transport of detrital kelp particles, dispersal distances for deposition to different depths were simulated under different current speeds for detritus with different sinking speeds. We calculated the time a particle would spend in the water column if it had 10 m vertical distance to sink (i.e. within the kelp forests) or 1000 m vertical distance to sink (i.e., transported off the shelf), which encapsulated the range of depths observations of kelp detritus^17,48^. We multiplied this timespan by horizontal water movement speeds of 0.05, 0.1 and 0.2 m s^−1^, which captures normally prevailing currents along the Norwegian coast (Norwegian Meteorological Institute, www.yr.no) and corresponded to the range of current speeds (0 to 0.5 m s^−1^ at 10 m depth) used by Gaylord *et al*.^27^ in their kelp spore dispersal model. These calculations do not reflect actual export, for that you need hydrographic models, but demonstrate expected export distances over a range of conditions.

## Electronic supplementary material

Video showing the measurement of kelp detritius sinking rates.

## Data Availability

The data sets generated during the current study are available from the corresponding author on reasonable request.
